# Ancestral Genome Inference Using a Genetic Algorithm Approach

**DOI:** 10.1371/journal.pone.0062156

**Published:** 2013-05-02

**Authors:** Nan Gao, Ning Yang, Jijun Tang

**Affiliations:** 1 Tianjin Key Laboratory of Cognitive Computing and Application, Tianjin University, Tianjin, China; 2 Department of Computer Science and Engineering, University of South Carolina, Columbia, South Carolina, United States of America; 3 School of Automation, Northwestern Polytechnical University, Shaanxi, Xi'an, China; Université Paris-Sud, France

## Abstract

Recent advancement of technologies has now made it routine to obtain and compare gene orders within genomes. Rearrangements of gene orders by operations such as reversal and transposition are rare events that enable researchers to reconstruct deep evolutionary histories. An important application of genome rearrangement analysis is to infer gene orders of ancestral genomes, which is valuable for identifying patterns of evolution and for modeling the evolutionary processes. Among various available methods, parsimony-based methods (including GRAPPA and MGR) are the most widely used. Since the core algorithms of these methods are solvers for the so called median problem, providing efficient and accurate median solver has attracted lots of attention in this field. The “double-cut-and-join” (DCJ) model uses the single DCJ operation to account for all genome rearrangement events. Because mathematically it is much simpler than handling events directly, parsimony methods using DCJ median solvers has better speed and accuracy. However, the DCJ median problem is NP-hard and although several exact algorithms are available, they all have great difficulties when given genomes are distant. In this paper, we present a new algorithm that combines genetic algorithm (GA) with genomic sorting to produce a new method which can solve the DCJ median problem in limited time and space, especially in large and distant datasets. Our experimental results show that this new GA-based method can find optimal or near optimal results for problems ranging from easy to very difficult. Compared to existing parsimony methods which may severely underestimate the true number of evolutionary events, the sorting-based approach can infer ancestral genomes which are much closer to their true ancestors. The code is available at http://phylo.cse.sc.edu.

## Introduction

With the increasing availability of fully sequenced genomes, we are now able to conduct genomic evolution study beyond the mere sequence level. Rearrangement of gene orders by operations such as reversal (also called inversion), transposition, fission, and fusion is known to be an important evolutionary mechanism. As these events are rare, they can be used to reconstruct evolutionary histories that extend far back in time [1]. Other than reconstructing deep evolutionary histories, another important application of genome rearrangement analysis is to infer gene order between ancestral and contemporary genomes. Such inference is valuable for identifying patterns of evolution and for modeling evolutionary processes (e.g. hot spots of rearrangement). As a result, genome rearrangement analysis has attracted a lot of attentions from biologists, mathematicians, and computer scientists [2], [3] since the pioneering paper of Sankoff [4].

Handling rearrangement events directly is mathematically very difficult: it took almost a decade to find the first polynomial algorithm that computes the reversal distance (i.e. the minimum number of reversal operations to transform one genome into another) [5], and it was just recently proved that the transposition distance is NP hard [6]. Yancopoulos et al. [7] proposed a simplified model that used the universal double-cut-and-join (DCJ) operation to account for all rearrangement events, which cuts a chromosome at two places and rejoins the four ends of the two cut places in a new way. Although there is no direct biological evidence for DCJ operations, these operations are very attractive because it provides a simpler and unifying model for genome rearrangement [8].

Main methods to infer ancestral gene orders are parsimony-based methods such as GRAPPA [9] and MGR [10]. The core of MGR and GRAPPA is to solve the median problems of *k* genomes, which is to find an ancestral genome that can minimize the sum of the pairwise distances between itself and each of the *k* given genomes.

**Figure 1:**
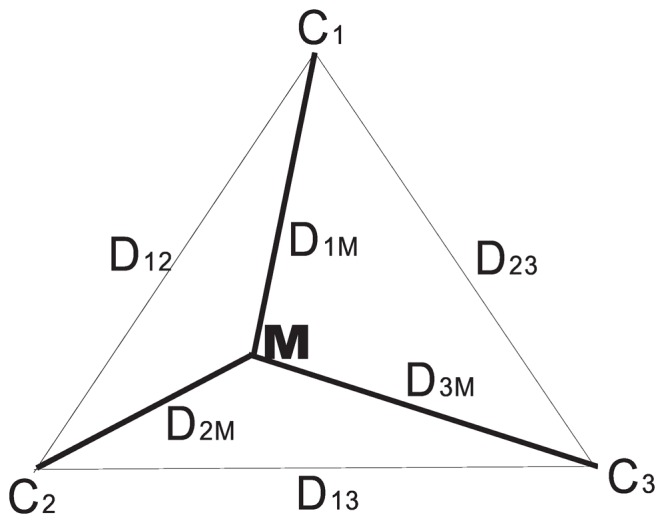
The DCJ median problem and its bounding box formed by the three outer edges.

The *DCJ median problem* is specifically defined as the problem to find a median genome that minimizes the summation of DCJ distances along the given three edges (Figure 1). It has been proved that this problem is NP-hard even for *three* genomes [11]. Because mathematically a DCJ distance is much simpler than handling events directly, parsimony methods using DCJ median solvers outperform other methods in terms of speed and accuracy. Among all existing exact solvers, the best is ASMedian proposed by Xu sand Sankoff [12], which uses the concept of Adequate Subgraph to decompose the problem into smaller, more easily solvable subproblems, thus significantly reduces the computation time. However it still runs very slowly when the genomes are distant. For datasets with *N* genes and *r* (expected) number of events per edge, when the ratio of *r*/*N* is larger than 50%, all median solvers have great difficulty in finishing the analysis after months of computation.

In this paper, we present a genetic algorithm (GA) which is based on sorting of two genomes to improve the DCJ median computation, with carefully designed procedures to produce the initial population and select individuals to create offspring. We have conducted extensive simulations and find that our GA-based method not only has better speed for difficult datasets, but also have better accuracy than existing methods.

## Background

### Genome Rearrangement Events

The popular way to represent a genome is to place genes on chromosomes, which is an ordering of genes with signs that indicate genomic strand. More formally, given a reference set of *n* genes 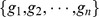, each gene is assigned with an orientation that is either positive, written as 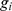, or negative, written as 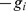. A chromosome can be linear or circular where its head meets its tail. In this paper, we assume that each gene appears exactly once in each genome.

Let *G* be the genome with signed ordering of 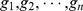. A *reversal* between indices *i* and *j* 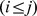, produces the genome with linear ordering 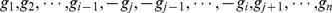. A transposition applies on three indices *i*, *j* and *k* (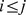) and produces the genome with linear ordering 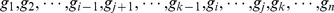 (assume 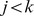).

If a genome has more than one chromosome, there are some additional events, such as translocation (the end of one chromosome is broken and attached to the end of another chromosome), fission (one chromosome splits and becomes two) and fusion (two chromosomes combine to become one).

Two genes *i* and *j* are called *adjacent* if *i* is followed by *j*, or −*j* is followed by −*i*. A *breakpoint* is defined when two genes are adjacent in one genome but not in the other. As we can represent a gene *g* by its tail 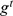 and its head 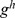, the adjacency of two consecutive genes *g*_1_ and *g*_2_ can have the following four types (depending on their respective orientation): 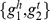, 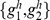, 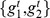, or 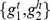. An extremity that is not adjacent to any other gene is called a *telomere*, represented by a singleton set 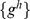 or 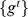.

### DCJ Distance and Sorting by DCJ

We can define the edit distance between two genomes as the minimum number of events to transform one into the other. The simplest distance is the breakpoint distance which is the number of adjacencies appears in one genome but not in the other.

The double-cut-and-join (DCJ) operation cuts the chromosome in two places and joins the four ends of the cut in a new way, in one of the following four cases [8]:

- A pair of adjacencies 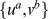 and 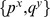 can be replaced by either the pair 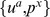 and 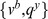 or the pair 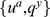 and 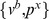.
- An adjacency 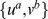 and a telomere 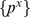 can be replaced by the adjacency 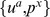 and telomere 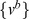 or by the adjacency 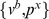 and telomere 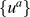.
- A pair of telomeres 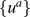 and 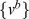 can be joined and replaced by the adjacency 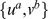.
- An adjacency 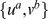 can be split and replaced by the pair of telomeres 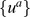 and 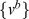.

**Figure 2:**
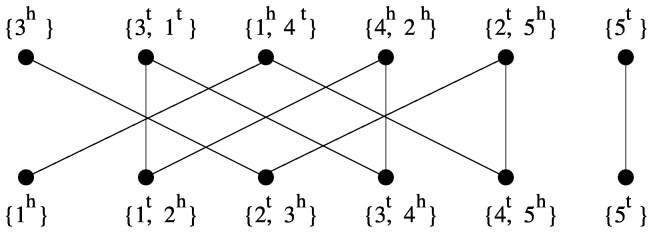
Adjacency graph and DCJ distance of two genomes *G*_1_ = (3,−1,−4,2,5) **and** *G*_2_ = (1,2,3,4,5)**.** The number of cycles *C* is 1, the number of paths *I* is 2, the DCJ distance is 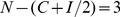.

Given two genomes *G*_1_ and *G*_2_, their DCJ distance can be computed using the adjacency graph 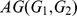, a graph whose set of vertices are the adjacencies and telomeres of *G*_1_ and *G*_2_. For each 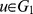 and 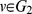 there are 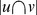 edges between *u* and *v* (Figure 2).

Based on the adjacency graph, we can easily compute the DCJ distance using the following formula [8]:
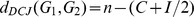



where C is the number of cycles and I is the number of odd edge paths in the adjacency graph 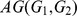 (Figure 2).

The adjacency graph is also the basic data structure in finding the optimal sequence of DCJ operations that transfers one genome into the other. There are two properties in the adjacency graph [8]: 1) no DCJ operation can simultaneously change the number of cycles C and the number of paths I; 2) for any pair of edges that connects two different vertices of *G*_1_ with an adjacency in *G*_2_, one single DCJ operation can transform them into a cycle of length two, and the remaining graph is reduced by the two edges. Thus this DCJ operation always increases 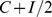 by one, making *G*_1_ one step closer to *G*_2_.

**Figure 3:**
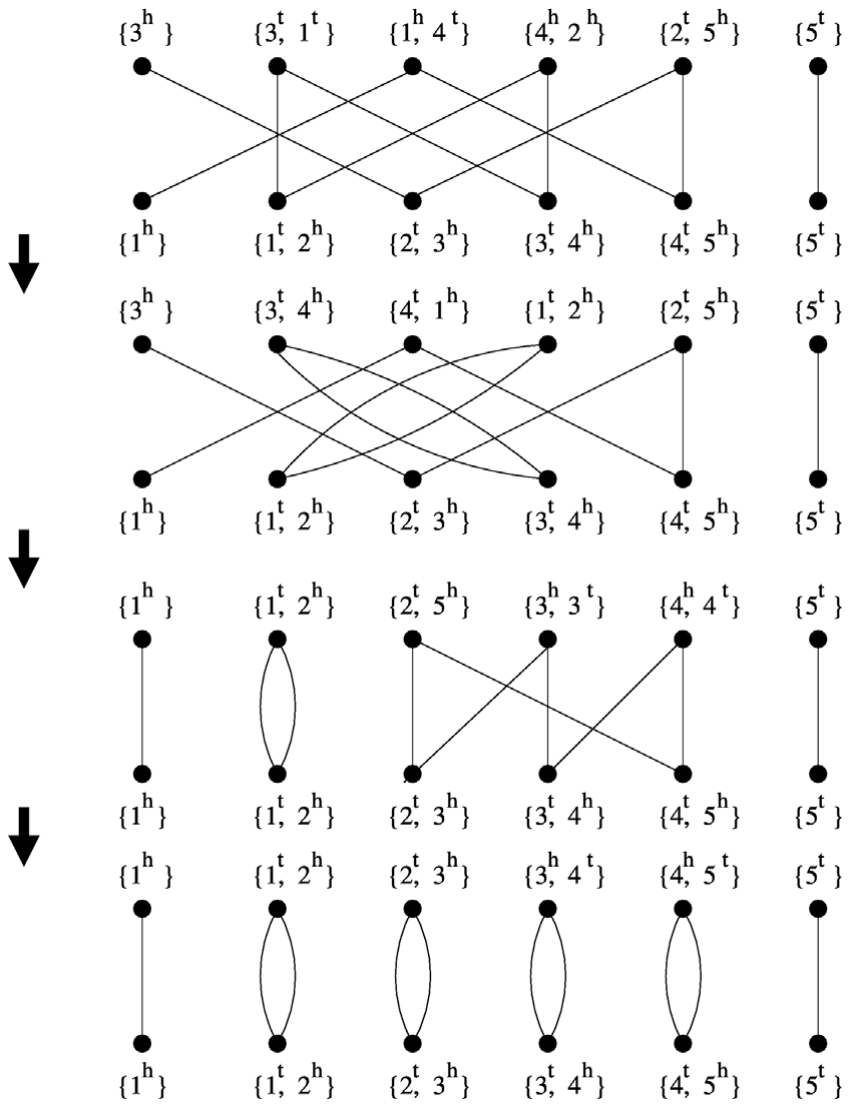
Adjacency graphs of each stage of one DCJ sorting sequence that transforms (3 −1 −4 2 5) to (1 2 3 4 5).

By greedily carrying out this operation, we can find one optimal sequence of events (sorting sequence) to transform *G*_1_ into *G*_2_. Figure 3 shows one example of three steps to transform genome *G*_1_ = (3,−1,−4,2,5) to *G*_2_ = (1,2,3,4,5). As there may be multiple pair of edges that have the above properties, the sorting sequence is not unique at all. For example, in Figure 3, the first step transforms *G*_1_ into a new genome (3,4,1,2,5); we can also transform *G*_1_ into (3,−1,−2,4,5), one step closer as well. If the genomes are distant, the number of all sorting sequences can be very large.

#### The DCJ Median Problem

In the DCJ median problem, given three genomes (leaves) *G*_1_, *G*_2_ and *G*_3_ and a genome *M*, the median score is defined as 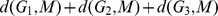, where *d* is the DCJ distance of two genomes. The aim is to find the genome with the minimum median score. As distances all abide by triangular inequality, we can define the perfect median score 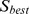 as.
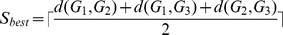
which is the lower bound of the median score (Figure 1). It should be pointed out that the median genome is not unique: there may exist many genomes with the same minimum median score and one may be closer to the ancestor than others. However existing methods all report the first best it encounters as the solution.

The median can be viewed as the ancestral genome that minimizes the evolutionary cost, thus finding medians forms the foundation of the most widely used genome rearrangement tools. For example, GRAPPA included Caprara's reversal median solver [13], as well as Xu and Sankoff's DCJ median solver (ASMedian). MGR uses a heuristic that seeks good reversals that bring a genome closer to the median genome. For datasets with more than three genomes, both will iteratively solve many instances of the median genomes to find a solution.

In ASMedian, the Multiple Breakpoint Graph (MBG) is used to model the median problem and an Adequate Subgraph is defined as the connected subgraph that has number of cycles larger or equal to 3*k*/4 (*k* is the number of the vertices of the subgraph). Once an adequate subgraph is found, the original MBG can be decomposed into two smaller MBGs thus the size of the original problem is reduced. Further decompositions could be performed iteratively to reduce the complexity of the problem. By searching existing adequate subgraphs, ASMedian can significantly reduce the computation time of median calculation. However, when the genomes are distant, the exhaustive search of adequate subgraphs requires not only long time but also very large amount of memory as ASMedian needs to store candidate subgraphs for further analysis.

Other than speed, the accuracy is also a concern as the DCJ median problem deals with edit distances and will severely underestimate the true number of evolutionary events. Different orders of searching and decomposing adequate subgraphs will result in different median solutions; however ASMedian does not take this into account and treats them in the order of their appearance. As a result, even the computation can finish, the inferred structures of ancestral genomes may not be trusted, which is confirmed by our experimental results. Through simulations, Haghighi and Sankoff [14] found that exact breakpoint median solvers have a tendency to place the median on or near of the leaves (the so-called “seeking corners” phenomenon), and conjectured that this should also be true for existing reversal and DCJ median solvers. As median computation forms the core of current genome rearrangement research, faster and more accurate methods are always desired.

### Genetic Algorithm

Genetic Algorithms (GA) [15], [16] were inspired by nature's robust way of evolution and also by Darwin's theory of natural selection: the fittest will have higher chance to survive.

For each generation, a genetic algorithm work on a population defined as a set of solutions (genomes in the DCJ median problem). It simulates the survival of the fittest individuals in the population, controlled by the definition of a fitness score. Generally an individual will be encoded into a sequence. Different sequences represent different solutions for the problem. Individuals in each generation are made to go through a process of evolution: selection by some fitness function, crossover with another individual, and randomly mutation at one or some spots in their encoding strings. Those individuals with the highest fitness score will be more likely to survive in each competition and will have higher chance to produce more offspring than those individuals that perform poorly.

With the evolution progresses, “good” gene segments will propagate throughout the population. Two good parents will have higher chance to produce better offspring than bad parents, as they have good gene segments. As a result, each successive generation will become more adapted to their living environment. A genetic algorithm will iterate until a solution is found or the maximum number of iterations is reached.

Genetic algorithms were widely used in solving many hard optimization problems, including those in computational biology [17]–[19]. Since genome rearrangement deals with chromosomes, evolutions and mutations, it will be natural to think that the approach of genetic algorithm can be easily adopted into solving the DCJ median problem. However, there are some major difficulties and the biggest problem is that the search space is simply too large: given genomes with *N* genes, the possible number of gene orders is 2^N^ *N*!. It poses serious questions on the major aspects of genetic algorithms: how should we generate the starting population, what is the best fitness score, and how to generate the next generation and pick the better one to survive?

## Methods

In this section, we present our sorting-based methods to tackle the major problems of using the genetic algorithm approach in the DCJ median problem. In this paper, an individual is simply a genome consists of linear or circular chromosomes, represented by the ordering of genes.

### Initial Population Generation

The initial population has deep impact on the performance of a GA-based method. In the DCJ median problem, as the search space is very large, randomly pick some genomes as start will not work as most likely these genomes will all be far away from the desired median, resulting in searches may not converge. Our approach is based on the following observation [20]: given three genomes, the median genome is likely to be on the path from one of the leaf genomes to another. Although this does not readily give us a median solver as the possible number of sorting paths are very large, it does suggest a strategy to generate the initial population: for any pair of the given genomes 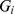 and 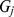 with distance 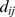, we will find genomes that are on the sorting path from 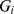 to 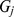 and are 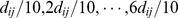 steps away from 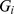. Such genomes on the sorting path can be easily generated using the DCJ sorting algorithm described earlier.

To obtain enough diversity, we generate 50 genomes per sampled step, resulting in 1,800 genomes in the initial population. As seen in the experimental results, this strategy is quite effective and sometime only a few steps are required to converge into very accurate results.

### Selection and Fitness Function

A critical parameter to be carefully tuned in GA is the selection pressure which is the process of selecting the best individual(s) for the next generation, governed by the fitness function. In the DCJ median problem, an obvious choice is to use the median score as the fitness function, and the one with a lower score will have better fitness. In practice, we use the following formula to calculate fitness scores: given *N* genes and the perfect median score 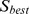, if a genome *G* has median score *S*, its fitness score is defined as
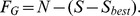



As the DCJ distance between any two genomes cannot exceed *N*, the above fitness function guarantees that the one closer to the median will be better, and the score is ranged between 0 and *N*.

In GA, an important step is to select individuals who can produce offspring–those having better fitness score should have higher chance to pass its good genes to the next generation. There are some classical mechanisms to select these individuals, based on different situations. For example, in Roulette Wheel Selection, each individual has its own probability of being selected into the candidate pool. One individual's probability is its fitness score divided by the sum of fitness scores of all individuals. Truncation Selection selects the top 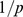 individuals and each will be copied *p* times into the pool.

In the DCJ median problem, the range of the fitness score is very small ([0,*N*]) compared to the possible number of genomes (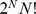), thus many individuals will have the same fitness score. This situation will get worse when the search approaches the end: the best candidate may have a fitness score that is only a few numbers away from the worst. Furthermore, two individuals with very different ordering of genes may have the same fitness score, but the difference of orderings may result in very different search directions: some may quickly converge to a good solution as they have better genomic structures while the others may not converge at all.

To overcome these problems, we adopt a hybrid approach of traditional selection methods. We first select the top 10% individuals and reproduce them (without change) into the next generation, as individuals with good genomic structure are hard to find and we want to preserve them as long as possible. We then give every individual in the remaining 90% an equal chance to be selected to produce offspring. To ensure better genes can be passed down, we devised the following crossover and mutation operations that are based again on genomic sorting.

### Crossover

Crossover is used for two selected individuals to exchange genetic materials and produce offspring. In some genetic algorithms, this procedure can be as simple as exchange blocks of their encoding strings. However, in the DCJ median problem, since each individual is represented as a gene order and each gene should appear exactly once in one individual, such exchange will result in invalid offspring.

The method we choose for crossover is based on sorting genomes by DCJ. First, we pick two parents (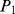 and 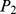) from the candidate pool and compare their fitness scores 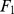 and 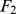. Assuming 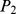 is better than 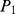, we will generate two children 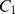 and 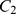. 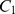 is generated by selecting a genome which is on the sorting path from 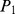 to 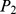 (the better one) and is *m* (randomly chosen) steps away from 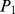. In other words, the new child obtains genetic materials from both parents by applying DCJ operations on one parent, with respect to the one with better fitness score. We do not generate 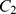 by sorting from the worse to the better, as from our experiments, this can easily destroy the good group of genes and lead to bad solutions. Instead, we generate 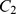 by directly copying of 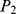 (which has better), giving the better genome a higher chance to pass its good structures into further generations. Both children will then undergo the mutation procedure described below with the expectation that better offspring may be found.

### Mutation

Mutation is used to maintain genetic diversities from one generation of a population to its next. Proper mutations are needed so that GA can avoid local minimal by preventing one population from becoming too similar to others.

In the DCJ median problem, an individual can be mutated by applying a random number of DCJ operations. However, there are two questions to be solved: how many operations are required and which operations should we choose to apply?

From Figure 1, one can *estimate* distances from the median to the three given genomes by the following simple calculations:
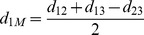


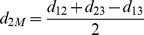


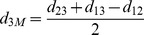



Although actual distances may be different from these estimated values, the above estimations are good indicators how close a candidate genome is from the median. If the three edge lengths of one genome are too far away from these relative estimated lengths, this genome is likely to be bad and should be mutated toward a better one.

Our mutation procedure is based on the above observation. For a genome *G*, we can compute its three edge lengths to the leaves, and find the one which has the largest differences from its estimate lengths. We then sort *G* some *m* (randomly chosen) steps closer to that leave.

We conduct the sorting-based mutation procedure on the two children genomes (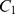 and 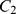) obtained from the above crossover procedure. As a result, we get two new genomes 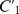 and 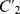. We then choose the two best from the four genomes (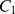, 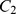, 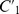 and 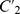), with the aim to maintain enough diversities and improve the quality of individuals in the next generation.

## Results

We implemented our new GA-based method in C and conducted experiments to assess its accuracy and speed. Simulation is the main approach to evaluate the quality of a phylogeny method, as its evolutionary history is known. In this paper, we conducted extensive simulations following widely accepted procedures. As ASMedian requires very large amount of memory when given genomes are distant, we used a shared-memory computer with 256GB memory to run the experiments, thus extended the range of problems that can be solved by ASMedian not normally achievable. Although the shared-memory computer is used, each test is done on a single CPU with no parallelism utilized.

### Setup of Simulations

Because all existing median solvers have very good performances when genomes are close but cannot finish when the genomes are distant, we divided our experiments into two parts: those can be finished by the exact methods and those cannot. We only compared our new GA method with Xu and Sankoff's ASMedian solver, as currently it is the best method of the DCJ median problem.

We tested the methods on simulated datasets. Each dataset has three genomes with 200 genes. We generated trees with three leaves and one internal node, assigned the identity permutation on the internal node and generated the three leaves by applying rearrangement events along each edge respectively. The number of events on each edge was controlled by a birth-death process which was viewed as a good model to fit evolutionary trees. The datasets were grouped by average edge lengths (*r*), which were 20 to 200 events per edge in our experiments, with the 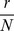 rates ranging from 0.1 to 1.0, representing data from very easy to extremely difficult. For each *r*, we generated 10 datasets and averaged the results.

The maximum number of iterations for our GA method was set as 500 but may stop early if the perfect median score was met. The genome with the lowest median score will be reported as the final result. In our experiments, this maximum number is large enough because we can find every best genome for every dataset within fewer than 500 iterations.

### Comparison with ASMedian

For *r*≤60ASMedian is generally very fast while our method is a bit slower. However, the running time of ASMedian increases quickly for *r*≥80 and requires more than a day to finish, while our GA method requires no more than 30 minutes even for the most difficult ones.

Table? 1 shows the results of obtained median scores. For *r*≤40, ASMedian and our method achieve the same median scores that are very close to the perfect median score. For *r*≥40, although the average median scores of our GA method are larger than those obtained by ASMedian, the differences are very small and less than 2% even for the most difficult cases. ASMedian could not finish any dataset in *r*≥140, while our method can still get genomes with reasonable median scores within 500 iterations and 30 minutes of computation.

**Table 1:** Comparison of median scores.

Comparison of the median scores (the lower the better):										
	r = 20	r = 40	r = 60	r = 80	r = 100	r = 120	r = 140	r = 160	r = 180	r = 200
Our GA Method	53.7	109.8	155.5	180.9	232.1	247.1	279.4	287.7	281.6	309.1
ASMedian	53.7	109.8	154.8	175.5	228	242.3	-	-	-	-
Perfect Score	53.6	109.4	152.2	173.4	210.6	221.8	242.4	254.8	244.4	261.9

*r* is the averaged number of events per edge. “-”indicates that a method cannot finish.

For the unrooted tree defined by the three given genomes, the median genome can be used to estimate the gene order of the internal node, which is the missing ancestor. Thus the distance to their true ancestor (known in simulations) is an additional measurement for the quality of median solvers. Table? 2 shows the average breakpoint distances to true ancestors for the two methods. It is very surprised to see for almost all datasets, the medians inferred by our GA method are indeed much closer to the true ancestors compared to those inferred by the exact method. This suggests that the sorting-based mutation and crossover procedures are very effective and preserve important genomic structures. Even for *r* = 200, the breakpoint distance between the inferred and true ancestor genomes is less than 55, comparable to those achieved by ASMedian for a far smaller *r* (*r* = 120 in Table? 3).

**Table 2:** Comparison of the breakpoint distance from the inferred median to the true ancestor.

Comparison to the true ancestors (the lower the better):										
	r = 20	*r* = 40	r = 60	r = 80	r = 100	r = 120	r = 140	r = 160	r = 180	r = 200
Our GA Method	0.3	0.4	5.0	9.9	28	32.7	44.9	49.2	57.5	54.9
ASMedian	0.4	0.3	6.3	15.6	40.7	50.5	-	-	-	-

*r* is the averaged number of events per edge. “-”indicates that a method cannot finish.

**Table 3:** Number of generations to find the best genome.

	*r* = 20	*r* = 40	*r* = 60	*r* = 80	*r* = 100	*r* = 120	*r* = 140	*r* = 160	*r* = 180	*r* = 200
Average	7.9	27.3	43	50.6	94.3	128.6	99.4	142.8	172.2	180.4
Max	21	104	108	110	201	290	151	303	337	496

### Convergence

An important aspect of GA is whether it can converge or not. Table? 3 shows the average and max number of generations our algorithm needed to find the best solutions. It is not surprising to see with higher number of events, the search space becomes much bigger, hence more generations are needed to get good genomes. It also tells us that although the maximum number of generations is set at 500, our GA method can always find good genomes with fewer generations. The average number of iterations is indeed much smaller than this upper limitation, thus better stop criteria may be desired to avoid this waste, using strategies such as checking population or genomic structure convergence.

## Conclusions

In this paper, we present a genetic algorithm for the DCJ median problem, a well-known problem in genome rearrangement analysis. Our GA-based method uses genomic sorting to generate initial populations and find offspring by crossover and mutations. Our experiments on simulated datasets show that this GA method is very efficient and has better speed and accuracy compared to other existing methods. It also confirms the importance of sorting in solving the DCJ median problem and avoid the phenomenon of seeking corners. Our approaches can be adopted to include other events such as deletions and insertions, for which linear algorithms are available to compute genomic distances, to further improve the ancestral inference from genome rearrangements. As we generally deal with many more genomes, we need to develop a genetic algorithm that can compute phylogenies and ancestors directly, without solving the median problem at all.
